# Molecular mechanisms of cell–cell spread of intracellular bacterial pathogens

**DOI:** 10.1098/rsob.130079

**Published:** 2013-07

**Authors:** Keith Ireton

**Affiliations:** Department of Microbiology and Immunology, University of Otago, Dunedin, New Zealand

**Keywords:** *Listeria*, *Shigella*, *Rickettsia*, actin-based motility, protrusions, cell junctions

## Abstract

Several bacterial pathogens, including *Listeria monocytogenes*, *Shigella flexneri* and *Rickettsia* spp., have evolved mechanisms to actively spread within human tissues. Spreading is initiated by the pathogen-induced recruitment of host filamentous (F)-actin. F-actin forms a tail behind the microbe, propelling it through the cytoplasm. The motile pathogen then encounters the host plasma membrane, forming a bacterium-containing protrusion that is engulfed by an adjacent cell. Over the past two decades, much progress has been made in elucidating mechanisms of F-actin tail formation. *Listeria* and *Shigella* produce tails of branched actin filaments by subverting the host Arp2/3 complex. By contrast, *Rickettsia* forms tails with linear actin filaments through a bacterial mimic of eukaryotic formins. Compared with F-actin tail formation, mechanisms controlling bacterial protrusions are less well understood. However, recent findings have highlighted the importance of pathogen manipulation of host cell–cell junctions in spread. *Listeria* produces a soluble protein that enhances bacterial protrusions by perturbing tight junctions. *Shigella* protrusions are engulfed through a clathrin-mediated pathway at ‘tricellular junctions’—specialized membrane regions at the intersection of three epithelial cells. This review summarizes key past findings in pathogen spread, and focuses on recent developments in actin-based motility and the formation and internalization of bacterial protrusions.

## 2. Introduction

Rapid microbial dissemination (‘spread’) within key host organs is a critical step in many infectious diseases. In the case of some intracellular bacterial pathogens, spread between human cells involves a phenomenon called actin-based motility (ABM) [1,2]. Bacteria that exhibit ABM include the enteric pathogens *Listeria monocytogenes* and *Shigella flexneri*, and select species of the arthropod-borne genus *Rickettsia* [1,2]. The hallmark of ABM is subversion of the host actin cytoskeleton to stimulate bacterial motility within a human cell. This intracellular motility ultimately leads to microbial spread between host cells.

**Figure 1.**
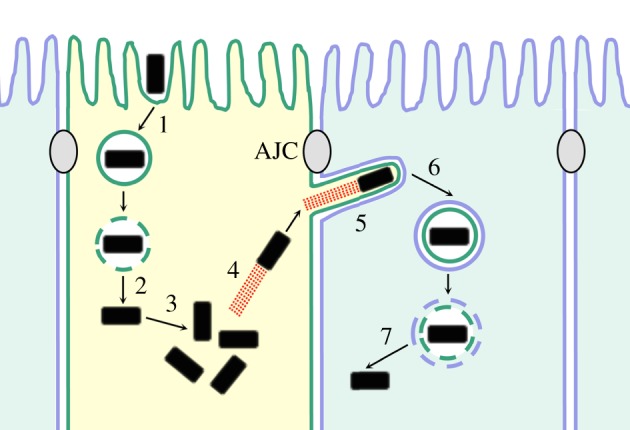
Steps in the intracellular life cycles of the bacterial pathogens *Listeria monocytogenes*, *Shigella flexneri* and *Rickettsia* spp. (1) internalization of bacteria into host cells, (2) destruction of phagosomes and access of bacteria to the host cytosol, (3) replication in the cytosol, (4) ABM, (5) formation of bacterial protrusions, (6) engulfment of protrusions and (7) dissolution of the double membranous vacuole. The process of cell–cell spread comprises steps 4–7. The initially infected cell that generates bacterial protrusions is coloured in yellow, whereas an adjacent cell internalizing a protrusions is blue. The plasma membranes of these cells are coloured differently in order to illustrate the origin of the two membranes in the vacuole resulting from protrusion engulfment (step 6). ‘AJC’ denotes the apical junctional complex—a structure composed of tight junctions and adherens junctions.

Cell–cell spread and other crucial steps in the intracellular life cycles of *Listeria*, *Shigella* and *Rickettsia* are depicted in figure 1 [1–7]. After internalization into human cells, bacteria are initially enclosed in host membranous structures called phagosomes (step 1). Within 30–60 min, phagosomes are destroyed through bacterial factors, allowing microbes access to the cytosol (step 2). Cytoplasmic bacteria replicate (step 3) and become decorated with host-derived actin filaments (step 4). Recruitment of F-actin is due to bacterial surface proteins that stimulate polymerization of actin monomers. The actin filaments organize into tail-like structures that produce ABM by propelling bacteria through the cytoplasm. Motile bacteria form protrusions derived from the host plasma membrane (step 5). These protrusions are ultimately internalized by surrounding host cells, resulting in bacteria encased in double membranous vacuoles (step 6). Bacterial enzymes destroy these vacuoles, liberating microbes and allowing infection of new human cells (step 7). For the purpose of this review, we define cell–cell spread as steps 4–7, starting with ABM and ending with escape from the double membranous vacuole. Throughout the spreading process, bacteria are encased in the plasma membrane of host cells. For this reason, spreading is thought to allow intracellular colonization of host tissues while shielding the pathogen from immune responses involving antibody or complement [6,8].

Over the past approximately 25 years, much progress has been made in understanding the molecular mechanisms of several aspects of the intracellular life cycles of *Listeria* and *Shigella*. In particular, bacterial and/or host proteins that mediate internalization, phagosomal escape, cytoplasmic replication, actin polymerization and destruction of the double membranous vacuole have been identified [3–7,9–33]. In many cases, how bacterial and/or human factors act at a molecular level to achieve these steps is partly understood. Research with *Rickettsia* has progressed more slowly compared with work with *Listeria* or *Shigella*, mainly because of difficulties in bacterial genetic analysis. However, recent results have shed light on modes of *Rickettsia* internalization and ABM [2,4,34–42]. Compared with steps 1–4 of the *Listeria*, *Shigella* or *Rickettsia* life cycles, the formation and engulfment of protrusions (stages 5 and 6) have proved more difficult to elucidate. Before 2005, it was unclear whether protrusions are generated simply as a passive consequence of ABM [43] or if instead mechanisms exist that act after bacterial-directed actin polymerization to directly govern protrusion formation [44]. In addition, whether uptake of protrusions involves active participation of the human cell was not understood. Recent findings identifying bacterial and host proteins that control protrusion formation [44–46] and human factors needed for engulfment of protrusions [47,48] represent important first steps in understanding post-ABM stages of bacterial spreading.

This review will cover molecular aspects of cell–cell spread of *Listeria*, *Shigella* and *Rickettsia*, focusing on ABM (step 4), protrusion formation (step 5) and protrusion internalization (step 5). Special emphasis will be given on findings obtained over the past 8 years. Other steps in the intracellular life cycles of these pathogens have been recently reviewed [2,4,6,7,34,49].

## 3. Actin-based motility

ABM of *Listeria*, *Shigella* or *Rickettsia* is promoted by bacterial surface proteins that are structurally distinct and stimulate actin polymerization through different means [1,2,5,49–51]. The *Listeria* protein ActA and *Shigella* protein IcsA both activate a mammalian actin polymerization machinery known as the Arp2/3 complex. The precise mechanisms of Arp2/3 activation by ActA or IcsA differ. The requirement for Arp2/3 in ABM of *Listeria* or *Shigella* indicates that these bacteria subvert an existing actin polymerization pathway in the human cell. By contrast, the *Rickettsia* protein Sca2 appears to directly stimulate assembly of actin filaments independently of Arp2/3 or other host factors. Sca2 may act as a functional mimic of a class of eukaryotic proteins called formins. In §3.1, I provide a brief summary of the mechanisms of actin assembly promoted by Arp2/3 or formins. For more extensive discussions of actin polymerization, the reader is referred to several recent reviews [50,52–54]. In §3.2, I describe actin assembly induced by *Listeria*, *Shigella* or *Rickettsia*, with emphasis on recent findings.

### 3.1. Mechanisms of actin polymerization in eukaryotic cells

#### 3.1.1. Actin filament assembly and function

Actin is present in a monomeric form, or as filaments derived from the polymerization of several actin monomers [50,54]. Actin filaments have a defined polarity, which determines the overall direction of filament growth. Actin monomers complexed with ATP tend to add on to the barbed (plus) end of an existing filament. ATP hydrolysis occurs in the filament, resulting in actin-ADP that ultimately dissociates from the pointed (minus) end of the filament. Addition of actin monomers on to the barbed end of a filament is enhanced by the protein profilin, which stimulates the exchange of ATP for ADP on monomers [55,56]. In cells, actin filaments are often dynamic, assembling or disassembling in response to external stimuli such as growth factors or extracellular matrix components [50,54]. The regulated assembly or disassembly of F-actin plays critical roles in many cellular processes. For example, actin polymerization helps remodel membranes, probably by generating force at actin–membrane interfaces [50,57]. This membrane remodelling function of F-actin contributes to many essential processes, including cell motility, cytokinesis, endocytosis and vesicular trafficking from the endoplasmic reticulum or Golgi apparatus [52,58]. Depending on the process, force generation may involve simply actin polymerization or the combined action of F-actin and myosin to produce contractility [58]. The controlled disassembly of actin filaments also impacts many important biological events, including regulated exocytosis [58] and the engulfment of particles through phagocytosis [59].

The first step in the de novo assembly of an actin filament is the formation of actin dimers or trimers [50,54]. This process, termed ‘nucleation’, is rate-limiting *in vitro*. In cells, several proteins exist that accelerate nucleation, thereby stimulating actin polymerization. These ‘nucleators’ fall into three general classes: the Arp2/3 complex, formin proteins and WH2 domain-containing nucleators [50,53,60–65]. Arp2/3- and formin-mediated nucleation are relevant to known mechanisms of bacterial ABM and are therefore discussed later. WH2 domain nucleators will not be covered in this review.

#### 3.1.2. Nucleation of actin filaments by the Arp2/3 complex or formins

**Figure 2.**
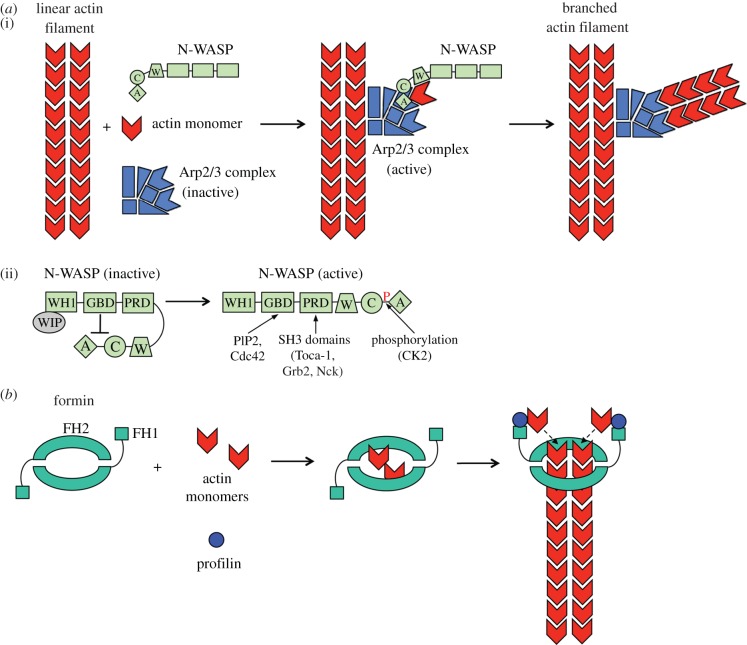
Actin polymerization mediated by the human Arp2/3 complex or formin proteins. (*a*) Arp2/3-dependent actin polymerization. (i) Cooperation of Arp2/3 and N-WASP to promote actin filament assembly. Arp2/3 is a seven-protein complex that stimulates the assembly of a new actin filament at the side of an existing filament [50]. Arp2/3-mediated F-actin assembly requires the participation of NPFs such as N-WASP. N-WASP uses its WCA domain to stimulate Arp2/3-mediated actin polymerization. The C and A regions of this domain bind and activate the Arp2/3 complex, whereas the W region delivers actin monomers to the Arp2/3 nucleation machinery. (ii) Regulation of N-WASP activity. N-WASP is subject to autoinhibition mediated by binding of its GTPase-binding domain (GBD) to the C and A regions. The protein WIP stabilizes the inactive conformation of N-WASP. Autoinhibition of N-WASP is relieved by binding of the phospholipid phosphatidylinositol 4,5-bis phosphate (PIP2), the activated GTPase Cdc42 or SH3 domains of several signalling proteins. In addition, N-WASP can be activated by phosphorylation of a serine residue in its WCA domain. (*b*) Formin-mediated actin polymerization. Formin proteins function as dimers and use two domains to stimulate the assembly of linear actin filaments [50,53]. The formin homology 2 (FH2) domain nucleates actin filaments, and the formin homology 1 (FH1) domain delivers profilin–actin complexes to the filament's barbed end. A, acidic region; C, connector region; CK2, casein kinase 2; SH3, Src Homology 3; W, WASP Homology 2 (WH2) domain; WIP, WASP-interacting protein.

Arp2/3 is an evolutionarily conserved complex of seven proteins [13,50,52,66–71]. Two of the seven components (Arp2 and Arp3) have structural similarity to monomeric actin [72]. The Arpc1 component has a WD40 domain that forms a seven-bladed beta propeller. The remaining components (Arpc2, Arpc3, Arpc4 and Arpc5) do not exhibit significant structural similarity to other known proteins. The Arp2/3 complex stimulates polymerization of a new actin filament from the side of an existing (‘mother’) filament, resulting in a Y-shaped branched actin structure [60,73,74] (figure 2*a*(i)). Studies involving electron tomography suggest that Arpc2 and Arpc4 contact the mother actin filament, whereas Arp2 and Arp3 interact with pointed end of the nascent filament. [75]. The Arp2 and Arp3 components are thought to form a dimer on the side of the mother filament, serving as the first subunits of the new actin filament [75,76]. Thus, the Arp2/3 complex may stimulate actin polymerization by mimicking an actin dimer, whose formation is normally the rate-limiting step in filament assembly.

By itself, the Arp2/3 complex is inefficient in promoting F-actin assembly. Efficient actin polymerization requires activation of the Arp2/3 complex by ‘nucleation-promoting factors’ (NPFs) [60,77–79]. One of the best characterized NPFs is neuronal Wiscott–Aldrich syndrome protein, or ‘N-WASP’ [80]. N-WASP uses a region called a WCA domain to activate Arp2/3 (figure 2*a*(i)). This domain interacts with the Arp2/3 complex [78], inducing conformational changes that bring the Arp2 and Arp3 components into close proximity and render the complex competent for nucleation [81–84]. In addition to activating the Arp2/3 complex, N-WASP also binds and delivers actin monomers to the nucleation machinery. The WCA domain interacts with monomeric actin, and a proline-rich region binds actin complexed with profilin [85,86].

N-WASP is itself subject to complex regulation (figure 2*a*(ii)) [50,80]. In the absence of cellular stimuli, N-WASP is autoinhibited due to intramolecular interactions that mask the activity of the WCA domain [78,87]. The protein WIP (WASP-interacting protein) stabilizes the inactive conformation of N-WASP [88]. In response to growth factors or other stimuli, autoinhibition of N-WASP is relieved through interactions with several cellular factors including the activated (GTP-bound) form of the small GTPase Cdc42, the lipid phosphatidylinositol 4,5-bis phosphate and Src Homology 3 domains from the signalling proteins Toca-1, Nck or Grb2 [78,89–94]. In addition to these regulatory interactions, activation of N-WASP is also promoted by serine phosphorylation of its WCA domain, which increases the affinity of this domain for the Arp2/3 complex [95].

In contrast to the branched actin structures produced by the Arp2/3 complex, formin proteins nucleate the assembly of linear actin filaments (figure 2*b*) [50,52,61,62]. The core elements common to all formins are the two ‘formin homology’ domains FH1 and FH2. Formins dimerize to form a ring-like structure that associates with the plus end of an actin filament [96–98]. The FH2 domains of formin dimers stimulate filament nucleation, probably by stabilizing actin dimers [99]. This domain also promotes elongation of actin filaments by preventing factors known as ‘capping proteins’ from halting polymerization [62,100,101]. The formin FH1 domain binds actin–profilin complexes, an action thought to increase the local availability of actin monomers for addition to the filament plus end [102,103].

At least 15 mammalian formins exist [50,53]. These proteins fall into seven different classes based on the FH2 domain amino acid sequence and the presence of additional domains that regulate FH1 and FH2 function [50,104]. The best-understood formin class is the diaphanous-related formins, comprising the proteins mDia1, mDia2 and mDia3 [50,53]. The FH1 and FH2 domains are located in the carboxyl-terminal region of Dia proteins. This region also contains a ‘diaphanous autoinhibitory domain’ (DAD) that regulates the actin polymerization activity of the FH2 domain. The DAD controls this activity by interacting with a ‘diaphanous inhibitory domain’ (DID) and ‘GTPase-binding domain’ (GBD) located in an amino-terminal region of Dia proteins. Binding of the DAD to the DID and GBD results in autoinhibition [105,106]. Upon cellular stimulation via engagement of cell surface receptors, autoinhibition is relieved by association of activated forms of the small GTPases Cdc42 or RhoA with the GBD of Dia proteins [107–109].

The Arp2/3 complex controls a variety of essential processes in mammalian cells, including endocytosis and membrane trafficking between the endoplasmic reticulum and Golgi apparatus [50,52,57]. Both Arp2/3 and diaphanous-related formins promote the formation of membrane extensions, called lamellipodia or filopodia, that drive cell motility, formation of cell–cell junctions and phagocytosis [50,52]. Given the ability of the Arp2/3 complex and Dia proteins to produce actin filaments that generate force and remodel cellular membranes, it is not surprising that many intracellular microbial pathogens have evolved mechanisms to exploit these two pathways of actin polymerization [2]. In §3.2, I explain how the bacteria *Listeria* and *Shigella* manipulate Arp2/3 or N-WASP in order to promote ABM. I also describe recent results indicating that *Rickettsia* stimulates intracellular motility by producing a bacterial mimic of eukaryotic formins. In §4, I provide further examples of bacterial subversion of Arp2/3 or formin function during protrusion formation leading to cell–cell spread.

### 3.2. Bacterial stimulation of actin assembly

#### 3.2.1. Listeria

*Listeria monocytogenes* is a Gram-positive food-borne pathogen capable of causing serious infections culminating in abortions or meningitis [3,110]. Cell–cell spread of *Listeria* is thought to be critical for disease, based on observations that bacterial mutants defective in spreading in cultured cells are compromised for virulence in a mouse animal model [20,111–113]. Sites of bacterial spread in infected animals, as indicated by histological studies, include the intestinal epithelium [114,115] and the liver [112]. Cell–cell spread not only facilitates colonization of key host organs, but also contributes to infection of the fetus in pregnant animals [116,117].

**Figure 3.**
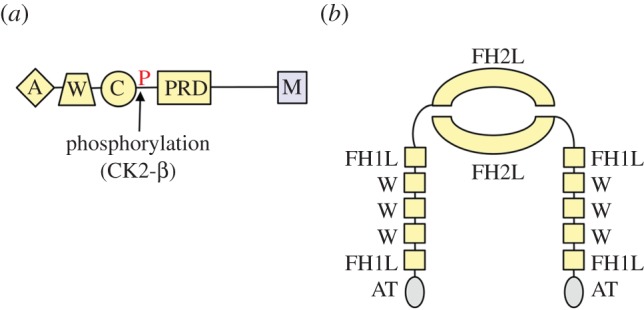
Bacterial surface proteins that stimulate actin polymerization. (*a*) Structure of the *Listeria* protein ActA. ActA is a bacterial NPF with structural and functional similarities to eukaryotic N-WASP [1,50]. An amino-terminal domain in ActA has sequences with amino acid similarity to the W, C and A regions in N-WASP. ActA's central proline-rich domain (PRD) serves a function similar to that of the PRD in N-WASP. A carboxyl-terminal membrane anchoring domain (M) is responsible for association of ActA with the bacterial surface. (*b*) Putative structure of the *Rickettsia* protein Sca2. Sca2 has regions with amino acid or secondary structural similarity to formin FH1 or FH2 domains, respectively [35,36]. Here, these regions in Sca2 are referred to as ‘FH1-like (FH1L)’ or ‘FH2-like’ (FH2L), respectively. Sca2 also has three WASP homology 2 (WH2) domains predicted to bind actin monomers. A carboxyl-terminal autotransporter (AT) domain in Sca2 is thought to anchor the protein to the outer membrane of bacteria [36]. Because of the sequence or structural similarity of Sca2 regions to domains in eukaryotic forms, Sca2 is depicted as a dimer. However, the true oligomerization state of Sca2 is not known.

ABM, the first step in *Listeria* spread, was first described nearly 25 years ago [8]. Since this discovery, seminal work from several research groups has partly elucidated the biophysics of ABM and identified the bacterial and host factors that contribute to this process. *Listeria* actively induces the polymerization of host actin filaments, a process that provides the driving force for cytoplasmic movement of bacteria [118]. The bacterial factor responsible for ABM is a surface protein called ActA [11,12]. Remarkably, ActA acts as a structural and functional mimic of the eukaryotic NPF N-WASP [1,2,51]. The amino-terminal domain of ActA contains sequences with amino acid similarity to C and A regions of N-WASP (figure 3*a*) [51,60,119]. This domain also has an actin monomer binding sequence that is a functional equivalent of the N-WASP W (WH2) region [119–121]. Like N-WASP, the amino-terminal domain of ActA activates the Arp2/3 complex, stimulating nucleation of branched actin filaments [60]. In addition to this amino-terminal domain, a central proline-rich region of ActA also contributes to ABM by binding the host protein VASP [122–126] One possible role of VASP is to recruit profilin, which promotes addition of actin monomers to the plus end of actin filaments [55,56,126,127].

It is noteworthy that ActA was the first protein, bacterial or eukaryotic, demonstrated to function as an NPF. In fact, work with ActA led to the discovery of the Arp2/3 complex as a machine that promotes the nucleation of actin filaments [13,60]. After these initial studies with ActA, N-WASP and other eukaryotic proteins were demonstrated to act as NPFs for Arp2/3 [77–79,128,129]. Thus, studies with ActA serve as a prime example of how microbial virulence proteins can be used as tools to understand fundamental aspects of eukaryotic cell biology.

In contrast to N-WASP, ActA lacks domains that mediate regulation by host GTPases. However, recent results indicate that ActA and N-WASP share a common regulatory mechanism involving serine phosphorylation in the vicinity of their C regions [130] (figure 3*a*). The C regions of N-WASP and another eukaryotic NPF called WAVE2 are immediately adjacent to consensus phosphorylation sequences for the eukaryotic serine/threonine kinase casein kinase 2 beta (CK2-β) [95]. Importantly, CK2-β-mediated phosphorylation of these sequences increases the affinity of N-WASP or WAVE2 for the Arp2/3 complex and enhances Arp2/3-dependent actin polymerization [95]. Strikingly, the C region in ActA contains adjacent CK2 consensus phosphorylation sites similar to those in N-WASP and WAVE2 [130]. A variety of biochemical and genetic approaches were used to demonstrate that CK2-β-mediated phosphorylation of one of these sites in ActA occurs *in vitro* and in infected human cells. Moreover, mutational analysis of ActA indicates that phosphorylation is needed for efficient interaction with the host Arp2/3 complex, bacterial ABM and full *Listeria* virulence in an animal model. These findings reveal that eukaryotic NPFs and the *Listeria* NPF ActA share a common post-translational regulatory mechanism. ActA can therefore be viewed not only as structurally and functionally mimicking eukaryotic NPFs, but also as exploiting the same host regulatory machinery. This latter facet has been termed ‘regulatory mimicry’ [130]. Since ActA and eukaryotic NPFs do not share extensive amino acid similarity except in their C regions and CK2-β phosphorylation sites, it has been suggested that these proteins arose through convergent evolution [130,131]. If so, then the shared regulation of eukaryotic and microbial NPFs by CK2-β would suggest that control of NPFs is critical, and a limited number of solutions exist for regulating C region activity.

#### 3.2.2. Shigella

*Shigella flexneri* is a Gram-negative bacterial pathogen that infects cells of the intestinal epithelium, resulting in dysentery [5]. Intracellular motility of *Shigella* was first described in the late 1960s [132], and movement was demonstrated to be actin-dependent about 20 years later [26]. Based on the work in an animal model, the ability of *Shigella* to undergo ABM and spread between intestinal epithelial cells is crucial for disease [133].

ABM of *Shigella* is induced by the bacterial surface protein IcsA, which is also known as VirG [26,27,29,30]. IcsA is unrelated in amino acid sequence to *Listeria* ActA, and these two bacterial proteins promote actin filament assembly through distinct mechanisms. Whereas ActA acts as a mimic of eukaryotic N-WASP, *Shigella* IcsA stimulates actin polymerization by using host N-WASP [1,49,51]. In infected human cells, N-WASP accumulates at the bacterial pole that produces the F-actin tail [28,134]. Bacterial recruitment of host N-WASP is due to IcsA, which uses an amino-terminal domain with glycine-rich repeats to bind directly to the human protein [28,134]. Importantly, N-WASP is essential for ABM of *Shigella*, as determined by experiments involving dominant negative N-WASP alleles or mouse cell lines deleted for the N-WASP gene protein [28,135,136]. *In vitro* experiments with purified proteins demonstrate that IcsA is capable of activating N-WASP, resulting in Arp2/3-dependent actin polymerization [134]. Interestingly, IcsA resembles the eukaryotic GTPase Cdc42 in its ability to activate N-WASP, suggesting that the bacterial protein could be considered a functional mimic of Cdc42. In agreement with this idea, ABM of *Shigella* is independent of Cdc42 [137,138].

A large proportion of cellular N-WASP is complexed with WIP, which stabilizes the autoinhibited form of N-WASP [80]. Cdc42-GTP alone is unable to activate N-WASP associated with WIP. Instead, activation requires the simultaneous presence of Cdc42 and an additional protein called Toca-1 [94]. Interestingly, WIP is recruited to motile *Shigella* [139], suggesting that bacteria need to overcome WIP-mediated inhibition of N-WASP in order to form F-actin comet tails. Consistent with this idea, recent results indicate a critical role for Toca-1 in ABM of *Shigella* [140]. Host Toca-1 associates with intracellular bacteria immediately prior to actin-based movement. Importantly, recruitment of Toca-1 is independent of IcsA and is instead mediated by an unidentified *Shigella* factor that is injected into the host cell through a bacterial apparatus termed a ‘type III secretion system’ [49]. Experiments involving RNAi-mediated depletion of Toca-1 indicate a crucial role for this human protein in bacterial-induced F-actin tail assembly [140]. Moreover, a constitutively activated derivative of N-WASP restores normal F-actin tail formation in cells depleted for Toca-1. This latter result suggests that Toca-1 controls *Shigella* ABM by contributing to N-WASP activation. Taken together with previous studies [28,134], these recent findings indicate that *Shigella* stimulates host Arp2/3-dependent actin polymerization by recruiting N-WASP via IcsA and exploiting Toca-1 to activate N-WASP.

#### 3.2.3. Rickettsia

The spotted fever group of *Rickettsia* cause severe systemic diseases characterized by infection of endothelial cells and increased microvascular permeability [4]. ABM and cell–cell spread of *Rickettsia* are thought to contribute to bacterial colonization of endothelial cells and resulting vascular dysfunction.

Interestingly, the F-actin tails of *Rickettsia* differ in structure from those of *Listeria* or *Shigella*. Whereas the latter bacteria have tails with a meshwork of branched actin filaments, *Rickettsia* tails comprise parallel bundles of linear actin filaments [1,141]. The linear filaments in *Rickettsia* tails suggest that this bacterium might not use the host Arp2/3 complex to induce actin polymerization. Indeed, several reports indicate that inhibition of Arp2/3 fails to affect ABM of *Rickettsia*, in contrast to the situation observed with *Listeria* or *Shigella* [142,143].

Recent results have shed light on how *Rickettsia* produces tails with linear actin filaments. The bacterial surface protein Sca2 was shown to be required for ABM [36] and to nucleate F-actin [35]. Interestingly, multiple lines of evidence indicate that Sca2 may be a structural and functional mimic of eukaryotic formin proteins. First, Sca2 has domains with structures and/or functions similar to regions in formins (figure 3*b*). Specifically, the amino-terminal domain of Sca2 is predicted to share secondary structure similarity with the FH2 domains of formins [35], and a central region in Sca2 has amino acid similarity to FH1 domains [36]. Sca2 also has three WASP homology 2 (WH2) domains that are predicted to bind actin monomers [35,36]. Second, the biochemical activities of Sca2 resemble those of formins [35]. Like formin proteins, Sca2 nucleates the assembly of linear actin filaments [35]. In addition, Sca2 promotes filament elongation by using profilin and inhibiting the activity of capping proteins. Importantly, Sca2 is needed for virulence in an animal model [36], demonstrating that ABM mediated by this bacterial protein contributes to disease.

It has been proposed that *Rickettsia* may have factors apart from Sca2 that contribute to ABM [1]. The *Rickettsia* protein RickA has amino acid similarity to the WCA region of N-WASP and is capable of stimulating Arp2/3-dependent actin polymerization *in vitro* [37,38]. However, a major function for RickA in ABM seems unlikely given the linear nature of actin filaments in *Rickettsia* tails [141], the absence of Arp2/3 in these structures [141] and the lack of requirement for Arp2/3 in *Rickettsia* motility in human cells [142,143]. It is possible that the major function of RickA is to induce actin cytoskeletal rearrangements involved in internalization of *Rickettisa* into host cells [35].

## 4. Bacterial protrusion formation

While much progress has been made in dissecting mechanisms of bacterial ABM, considerably less is understood about the subsequent steps of membrane protrusion formation and engulfment. A key issue in the field has been whether pathogen-containing protrusions develop simply as a passive consequence of actin-based movement or whether instead these structures are actively controlled by bacterial and/or host factors [43]. Although this area of research is in its infancy, recent results suggest an active involvement of pathogen and host in the generation of protrusions [44–46]. Key findings with *Listeria* and *Shigella* are described later. At present, studies on mechanisms of protrusion production by *Rickettsia* have not been reported.

### 4.1. Listeria

Recent results have led to the identification of *Listeria* and human proteins that control the generation of bacterial protrusions in polarized human intestinal epithelial cells. After internalization into host cells, cytoplasmic *Listeria* secretes a protein called InlC [144] that acts after F-actin tail assembly to enhance protrusion formation and cell–cell spread [45]. InlC promotes protrusions by physically interacting with and antagonizing the function of a human cytoplasmic protein called Tuba. Tuba is a large scaffolding protein with several functional domains, including a carboxyl-terminal Src Homology 3 (SH3) domain that associates with human N-WASP. Biochemical data demonstrate that InlC binds directly to the Tuba SH3 domain, thereby displacing N-WASP. Experiments with *Listeria* expressing a mutant InlC protein defective in binding Tuba indicate that the ability of InlC to displace N-WASP is critical for bacterial spread in cultured cells. Altogether, these findings demonstrate that *Listeria* enhances its spreading by disrupting complexes composed of host Tuba and N-WASP.

Studies on the role of InlC in spread have recently been extended to an animal model [112]. This work took advantage of a mutant InlC protein that folds normally but is compromised in binding to the Tuba SH3 domain. *Listeria* expressing this mutant InlC protein has a virulence defect in intravenously inoculated mice that is similar to the defect of an *inlC* deletion strain. In addition, the *Listeria* mutant strain producing InlC defective in binding Tuba exhibits decreased cell–cell spread in the mouse liver. These studies support the idea that the ability of InlC to interact with host Tuba is important for *Listeria* virulence.

**Figure 4.**
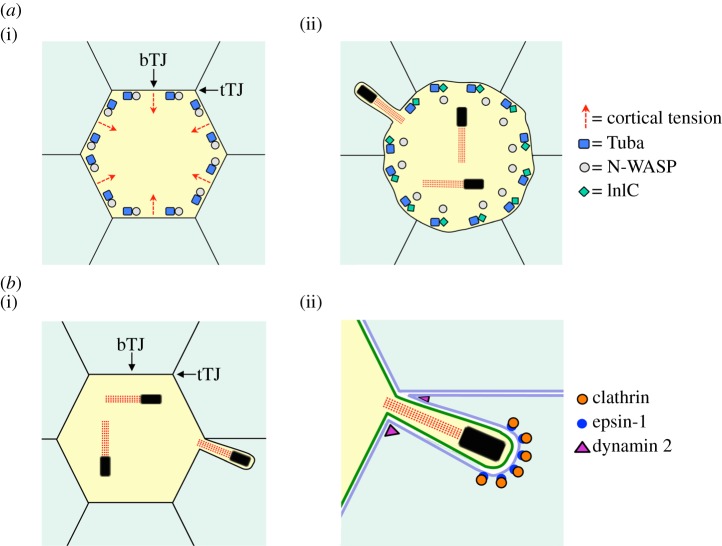
Spreading of *Listeria* and *Shigella* involves remodelling of host cell–cell junctions. (*a*) The formation of *Listeria* protrusions is enhanced by bacterial-induced alterations of tight junctions (TJs). (i) The human proteins Tuba and N-WASP form a complex that helps maintain linear TJs, probably by generating cortical tension at the plasma membrane [45,145]. bTJ and tTJ denote ‘bicellular tight junction’ and ‘tricellular tight junction’, respectively, which are formed by the intersection of two or three cells, respectively. (ii) In human cells infected with *Listeria*, the secreted bacterial protein InlC binds to host Tuba, thereby disrupting Tuba–N-WASP complexes [45]. This inhibition of Tuba and N-WASP results in slack junctions, which are likely to reflect diminished cortical tension. The relief in tension is thought to facilitate bacterial spread by removing an inward force at the host plasma membrane that would otherwise counteract the outward force exerted by motile bacteria. (*b*) *Shigella* protrusions are internalized through a host endocytic pathway at tTJs. (i) Live imaging studies indicate that *Shigella* contact with tTJs leads to productive spreading [48]. (ii) tTJs affect the internalization of bacterial protrusions. Several human proteins known to promote clathrin-mediated endocytosis (clathrin, epsin-1, dynamin 2) are needed for engulfment of *Shigella* protrusions, suggesting that bacteria may usurp a host endocytic pathway to facilitate their spread. Epsin-1 and clathrin are depicted as accumulating in coated pits in the host cell receiving the protrusion, and dynamin 2 is shown mediating scission of the protrusion. These activities of epsin-1, clathrin and dynamin 2 are speculative.

What are the normal functions of human Tuba and N-WASP, and how does antagonism of these two host proteins by bacterial InlC enhance *Listeria* spread? In epithelial cells, Tuba and N-WASP act together to control morphology of the apical junction complex [145]—a structure composed of tight junctions and adherens junctions [146]. Adherens junctions promote cell–cell adhesion, whereas tight junctions act as a barrier to limit permeability to macromolecules and ions [146,147]. Tight junctions also contribute to cell polarity by establishing apical and basolateral plasma membrane domains. Importantly, RNAi-mediated depletion of Tuba or N-WASP causes tight junctions to become slack, suggesting a loss of cortical tension [45,145]. Thus, one of the normal functions of Tuba/N-WASP complexes is in the maintenance of proper junctional structure, possibly by generating tension through actin polymerization (figure 4*a*(i)). Interestingly, infection with *Listeria* or ectopic expression of InlC causes tight junctions to slacken, similar to the effects of Tuba or N-WASP depletion [45] (figure 4*a*(ii)). These findings indicate that InlC perturbs cell–cell junctions, probably through inhibition of Tuba and N-WASP. Altogether, the results suggest that Tuba/N-WASP complexes impose a potential barrier to bacterial spread by generating tension at cell junctions. This tension is expected to limit spreading by opposing the protrusive force of motile bacteria. By producing InlC, *Listeria* has evolved a mechanism to counteract the host machinery that normally generates cortical tension.

Apart from Tuba and N-WASP, another host factor that contributes to *Listeria* protrusion formation is the cytoskeletal regulatory protein ezrin [44]. Ezrin is a member of the ERM (ezrin/radixin/moesin) family of proteins [148]. ERM proteins possess a carboxyl-terminal domain that interacts with F-actin [149,150] and an amino terminal domain that binds to plasma membrane-associated proteins [151,152]. These domains provide ERM proteins with the ability to link the actin cytoskeleton to the plasma membrane. ERM protein activity is subject to autoinhibition mediated by interaction of the amino- and carboxyl-terminal regions [148]. One of the ways that autoinhibition is relieved is through phosphorylation of key threonine residue (T567) in the carboxyl-terminal domain of ERM proteins [153–155]. Importantly, the ERM protein ezrin localizes to *Listeria* F-actin tails in protrusions, but not to tails in the main body of infected cells [44,141,156]. Inhibition of ERM proteins through RNAi or expression of dominant negative ezrin alleles inhibits the formation of *Listeria* protrusions [44]. Experiments with an ezrin protein mutated in the T567 site indicate that activation of ERM proteins through phosphorylation is crucial for bacterial spread. Collectively, these results demonstrate an important role for host ERM proteins in the generation of *Listeria* protrusions. Interestingly, conditions that impair ERM protein function not only reduce the number of bacterial protrusions per host cell, but also alter the morphology of the few protrusions that are made. Compared with *Listeria* protrusions made under normal conditions, protrusions formed in cells with reduced ERM protein activity are shorter and wider, particularly in the region where the protrusion joins the main body of the cell. This aberrant morphology is consistent with the idea that ERM proteins may confer rigidity to *Listeria* protrusions by cross-linking F-actin tails to the host plasma membrane. This rigidity could contribute to cell–cell spread by allowing bacteria initiating protrusions to resist inward tension at cell junctions.

### 4.2. Shigella

Specific bacterial factors involved in the formation of *Shigella*-containing protrusions have not been identified. Interestingly, the same bacterial type III secretion system that promotes internalization of *Shigella* into host cells is also needed for cell–cell spread [157]. As mentioned in §3.2.2, one of the ways that this type III secretion system controls spreading is by stimulating F-actin tail assembly through recruitment of Toca-1 [140]. Whether the secretion system also acts after ABM to directly affect protrusion formation is not known. It would be interesting to screen known bacterial substrates of this secretion system for roles in the generation of bacterial protrusions.

Host factors that promote the formation of *Shigella-*containing protrusions include formin proteins, the actin-dependent motor protein myosin X and the cell–cell adhesion molecule E-cadherin. The role of formins appears to be in stimulating the assembly of actin filaments in comet tails in protrusions [158]. Thus, while *Shigella* F-actin tail formation in the main body of the cell requires host N-WASP and the Arp2/3 complex, actin polymerization in protrusions is thought to switch to a formin-mediated pathway. One of the key findings in this study is that the diaphanous formin Dia1 localizes to F-actin tails in the protrusions, but not to those in the cell body [158]. In addition, inhibition of Dia1 or Dia1 formins through RNAi or dominant negative approaches reduces the frequency of protrusion formation by *Shigella*. It was proposed that the switch to formin-mediated actin polymerization facilitates bacterial protrusion generation by re-organizing the dense cortical actin network that underlies the plasma membrane [158]. If not remodelled, this network would be expected to limit contact of motile bacteria with the plasma membrane. A second role of formin-mediated actin polymerization in comet tails may be to generate force necessary to deform the plasma membrane into protrusions.

Myosin X is a ubiquitously expressed unconventional myosin involved in filopodia formation and phagocytosis [159]. A recent report demonstrates an important role for this myosin in the formation of *Shigella* protrusions leading to cell–cell spread [46]. Myosin X is recruited to *Shigella* F-actin tails in protrusions [46]. RNAi-mediated depletion of myosin X reduces the length of *Shigella* protrusions, without affecting the number of protrusions produced per infected cell. These findings indicate that myosin X is dispensable for the initiation of protrusions, but required for their extension. Myosin X has several domains, including a head domain with ATP-dependent motor activity and a Pleckstrin homology (PH) domain that associates with the inner face of the plasma membrane [159]. The ability of myosin X to extend protrusions requires both of these domains [46]. Interestingly, time-lapse microscopy indicates that myosin X localization to protrusions is dynamic, with myosin clusters in F-actin tails cycling towards or away from protrusion tips. This cycling may reflect the movement of myosin motors along the actin filaments in tails. Based on these data, a model was proposed whereby the head domain of myosin X interacts with bacterial F-actin tails in an emerging protrusion and the PH domain associates with the plasma membrane [46]. According to this model, ATP-dependent motor activity causes myosin X to ‘walk’ along F-actin filaments in the tail, in the direction of filament plus ends. Since the myosin is anchored to the host plasma membrane, its directional movement along F-actin tails stimulates translocation of membrane towards the protrusion tip, driving protrusion growth. While attractive, this model may at first glance seem at odds with the observation in time-lapse microscopy that myosin X sometimes moves towards the base of protrusions [46]. Perhaps this movement is a recycling process that allows the same clusters of myosin X to be used multiple times for protrusion growth.

Interestingly, in addition to decreasing *Shigella* protrusion length, depletion of myosin X increases width in the region where protrusions intersect the main body of the cell [46]. This effect on *Shigella* protrusion width shows striking similarity to the role of ERM proteins in controlling the width of *Listeria* protrusions. These findings suggest that a second function of myosin X may be to confer rigidity to protrusions by linking F-actin tails to the plasma membrane.

An early study demonstrated an important role for host E-cadherin in the generation of *Shigella* protrusions of proper structure and also in the internalization of these protrusions by neighbouring host cells (see §5) [160]. This study used fibroblast cell lines lacking or expressing chicken E-cadherin. Scanning and transmission electron microscopy analysis indicated that protrusions made in cells lacking E-cadherin were flaccid, lacking tight association of bacteria and F-actin tails with the host plasma membrane. In addition, immunofluorescence studies revealed the presence of adherens junction components in F-actin tails in protrusions. Interestingly, these comet tails appear to intersect cell–cell junctions, suggesting that protrusions emanate from adherens junctions. Collectively, the results in this study indicate the E-cadherin-mediated cell–cell adhesion is essential for *Shigella* spread, and that bacterial protrusion formation may involve targeting of adherens junctions.

## 5. Internalization of protrusions

An important question is whether *Listeria*, *Shigella* or *Rickettsia* produce factors that stimulate the ability of host cells to engulf protrusions. Thus far, such microbial factors have not been identified. In recent years, some progress has been made in identifying host proteins specifically involved in the internalization of *Listeria* or *Shigella* protrusions. These findings are discussed later.

### 5.1. Listeria

An early study proposed that engulfment of *Listeria* protrusions might occur through a host-driven process that is active even in the absence of microbial infection [161]. This proposal was based on the observation that vesicles produced by labelled uninfected epithelial cells were internalized by neighbouring cells [161]. The overall size and shape of these vesicles resembled those observed during cell–cell spread of *Listeria*. The process of membrane internalization between neighbouring cells was termed ‘paracytophagy’.

Despite these interesting early observations, progress in identifying human factors mediating engulfment of *Listeria* protrusions has been slow. So far, only one such factor has been found—human casein kinase 1 alpha (CK1-α) [47]. Identification of CK1-α was accomplished through a high-throughput RNAi-based screen in the human cell line HeLa. This screen involved assessing the roles of 779 known human kinases in cell–cell spread of *Listeria*. After performing experiments to exclude off-target effects of siRNAs, two of the original approximately 800 kinases investigated were found to have bona fide roles in spreading. These kinases were CK1-α and casein kinase 2 beta (CK2-β). As described in §3.2.1, CK2-β controls the actin polymerization step of *Listeria* spread through phosphorylation of ActA. Further analysis of CK1-α indicated that this kinase is dispensable for F-actin comet tail formation and the formation of protrusions, but is needed for the resolution of protrusions into vacuoles containing *Listeria*. Interestingly, CK1-α controls bacterial spread by acting in the infected host cell that donates the protrusion, not in the neighbouring cell that receives the protrusion. How CK1-α accomplishes protrusion resolution is not known, and solving this mystery may require additional RNAi-based screens of known casein kinase 1 substrates. It is possible that CK1-α phosphorylates a human protein involved in plasma membrane scission. This idea is discussed further in §6.

### 5.2. Shigella

As mentioned in §4.2, E-cadherin is required not only for the formation of *Shigella* protrusions of normal morphology, but also for the internalization of these structures by neighbouring cells [161]. It is not currently known if there is a direct role for E-cadherin or other adherens junction components in uptake of protrusions or whether the internalization defect in E-cadherin deficient cells is a secondary consequence of the aberrant protrusions formed. In addition to adherens junctions, gap junctions have also been implicated in the intercellular dissemination of *Shigella* [162]. The gap junction component connexin 26 was found to enhance the spreading of *Shigella* through a mechanism involving the release of ATP into the external medium. The specific stage of spreading affected by connexin 26 (e.g. F-actin tail formation, protrusion formation, protrusion engulfment) was not addressed. Altogether, these findings highlight the importance of cell–cell junctions in *Shigella* spread, a general feature now known to be shared with *Listeria* [45].

A recent study has investigated the role of host junctions in *Shigella* spread in further detail. Using time-lapse microscopy, *Shigella* was found to spread predominantly through contact with tricellular tight junctions (tTJs) [48], areas in an epithelial cell monolayer formed by the intersection of three cells (figure 4*b*(i)) [163]. By comparison, bicellular tight junctions (bTJs) are defined by the intersection of two cells. Despite the fact that tTJs occupy a very small area relative to bTJs, approximately 80% of cell–cell spread events with *Shigella* occur through tTJs [48]. These findings demonstrate that tTJs are membrane contact sites allowing highly efficient spread of *Shigella*. About 40–50% of *Listeria* spreading events occur at tTJs, indicating a lesser but still significant role for tTJs in dissemination of this pathogen [48]. An important component of tTJs is the protein tricellulin [163,164]. Tricellulin localizes predominantly to tTJs and is also found in lesser abundance at bTJs. Importantly, RNAi-mediated depletion of tricellulin impairs cell–cell spread of *Shigella*, indicating a functional role for tricellulin in this process [48]. In addition, spreading of *Shigella* through tTJs requires several human proteins with known roles in endocytosis, including epsin-1, clathrin and dynamin 2 [48]. Interestingly, depletion of tricellulin or these endocytic proteins does not affect the number of *Shigella* protrusions formed per cell, suggesting that these proteins mediate protrusion engulfment (figure 4*b*(ii)). In support of this idea, fluorescence microscopy analysis of live infected human cells reveals recruitment of endocytic proteins in cells internalizing protrusions [48].

Why does *Shigella* spread occur predominantly at tTJs? One possibility is that tTJs are regions of high endocytic activity, a property that would explain the apparent preference for internalization of protrusions in these regions. This idea is pure speculation, since to the best of my knowledge endocytosis at tTJs has never been investigated. It is worth remarking that tTJs create a channel or ‘central tube’ approximately 10 nm wide in the epithelial monolayer [163]. The plasma membrane surrounding this channel might have a protein and/or lipid composition distinct from that in bTJs—differences that could potentially affect many processes, including endocytosis.

## 6. Conclusions and outstanding questions

Since the first description of cell–cell spread nearly 25 years ago, the molecular basis of F-actin assembly by *Listeria* and *Shigella* has been extensively characterized to the point where it is possible to reconstitute ABM with purified bacterial and host proteins [165]. Substantial progress has recently been made on the mechanism of ABM by *Rickettsia* [35,36,143]. Studies on *Listeria*, *Shigella* and *Rickettsia* have revealed that ABM occurs through exploitation of host proteins (e.g. N-WASP and Arp2/3) and/or the action of bacterial proteins (ActA and Sca2) that structurally and functionally mimic eukaryotic NPFs or formins [11–13,26–30,35,36,134]. Interestingly, *Listeria* or *Shigella* subvert host regulatory mechanisms involving serine phosphorylation or the N-WASP activator Toca-1 to promote F-actin assembly [130,140]. The molecular bases of bacterial protrusion formation and engulfment are less well understood than ABM and have been the subject of several recent studies. Findings with *Listeria* and *Shigella* indicate that bacteria target host cell–cell junctions to facilitate the generation or the internalization of protrusions [45,48]. In addition, the formation of *Shigella* protrusions involves myosin motor activity [46] and a switch from Arp2/3- to formin-mediated actin assembly [158]. These recent findings on bacterial protrusions prompt a variety of important questions to address in future work. Some of these questions are outlined as follows.

**Figure 5.**
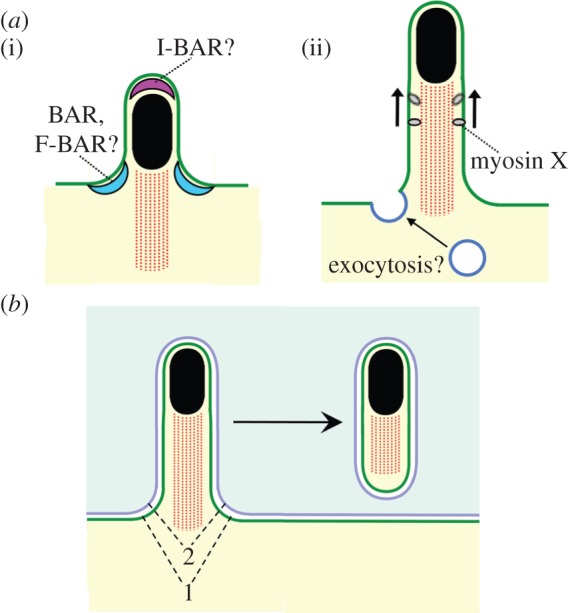
Potential mechanisms controlling the formation and engulfment of bacterial protrusions. (*a*) Formation of protrusions. (i) Remodelling of the host plasma membrane during the initiation of protrusion formation requires generation of negative and positive curvature at the protrusion tip and base, respectively. Several classes of human BAR proteins capable of producing negative or positive curvature exist [57,172]. I-BAR proteins have convex plasma membrane-binding domains and may help to remodel membrane at protrusion tips. BAR and F-BAR proteins have concave membrane-binding domains and could act the base of protrusions. (ii) Extension of protrusions. The motor protein myosin X promotes the growth of *Shigella* protrusions, possibly by transporting host plasma membrane towards the protrusion tip [46]. An unresolved question is whether extension of bacterial protrusions also requires the localized delivery of new host membrane through exocytosis. (*b*) Engulfment of protrusions. Internalization and conversion of a protrusion to a double membranous vacuole requires the scission and rejoining of plasma membrane in the human cell donating the protrusion (i) and also in the cell receiving the protrusion (ii). Host or bacterial factors responsible for these two scission/rejoining events are yet to be identified.

— *Does protrusion formation by *Listeria* involve a transition to formin-mediated actin polymerization*? Interestingly, *Listeria* F-actin tails in protrusions consist predominantly of linear filaments, in contrast to the branched F-actin network in tails in the host cell body [156]. The linear nature of filaments in protrusions raises the possibility that *Listeria*, like *Shigella* [158], undergoes a switch to host formin-mediated actin polymerization.
— *What host cell process is antagonized by *Listeria* to perturb apical junctions?* The *Listeria* protein InlC alters the structure of apical junctions by inhibiting human Tuba and N-WASP [45]. Elucidating the mechanism by which InlC affects junctions will first require understanding how Tuba and N-WASP normally control junctional structure. These two human proteins could directly affect junctions through the generation of F-actin involved in actomyosin-mediated tension [166]. Alternatively, Tuba and N-WASP might indirectly impact junction morphology through control of membrane trafficking pathways. For example, N-WASP promotes endocytosis [50,57] and vesicular trafficking at the Golgi apparatus [167–169]. Tuba is localized at sites of endocytosis and the Golgi [170,171], raising the possibility that this protein aids N-WASP in membrane trafficking.
— *Does cell–cell spread of *Shigella* and/or *Rickettsia* involve relief of tension at cell–cell junctions?* Similar to *Listeria*, *Shigella* infects polarized cells of the intestinal epithelium [5]. These epithelial cells contain AJs and TJs subject to cortical tension [147,166]. *Rickettsia* spreads in human endothelial cells [4], another polarized cell type with AJs and TJs [147]. An interesting question is how *Shigella* and/or *Rickettsia* cope with cortical tension at junctions. Have these pathogens evolved strategies similar to *Listeria*'s ability to dissipate tension?
— *How does host membrane remodelling occur at bacterial protrusions?* Protrusions have negative curvature at their tip and positive curvature at their base (figure 5*a*(i)). How is the plasma membrane of the host cell reshaped to form bacterial protrusions? Remodelling of eukaryotic membranes into curved shapes is accomplished by a variety of membrane-bending proteins of the BAR domain superfamily [172]. Some BAR domain proteins have convex membrane-binding surfaces, allowing them to induce negative membrane curvature. Other BAR domain proteins use concave surfaces to impart positive curvature to membranes. Importantly, several BAR proteins with convex or concave membrane-binding surfaces promote the formation of filopodia in mammalian cells [57]. Filopodia are plasma membrane projections that superficially resemble bacterial protrusions, but are of smaller diameter. In future work, it will be interesting to investigate the role of these BAR domain proteins in protrusion formation by *Listeria*, *Shigella* or *Rickettsia*.
— *How do bacterial protrusions extend?* Experiments with *Shigella* suggest that myosin X may promote the growth of protrusions by transporting host plasma membrane towards the protrusion tip [46]. Could protrusion extension also involve the local insertion of new host membrane delivered from intracellular compartments? Apical junctions, structures exploited by *Listeria* and *Shigella* for spread, are active sites of exocytosis [146,173–175]. Interestingly, these junctions are located in close proximity to the exocyst [176]—a multicomponent machinery that promotes tethering and fusion of intracellular vesicles with the plasma membrane [177]. An intriguing idea is that bacterial pathogens might exploit host exocytic activity at junctions to provide membrane needed for protrusion growth (figure 5*a*(ii)).
— *Does internalization of protrusions involve exploitation of host endocytic pathways that normally control junctional integrity?* Apical junctions are sites of endocytosis, as well as exocytosis [146,173,174]. Constitutive endocytosis of TJ and AJ components is thought to be involved in epithelial tissue homeostasis [146]. An important question is whether bacterial pathogens hijack junctional endocytic routes to allow internalization of protrusions. In this regard, it would be interesting to determine the extent to which the endocytic pathway that promotes engulfment of *Shigella* protrusions [48] also affects internalization of TJ and AJ components in healthy epithelia. In addition, it will be important to assess the roles of known host endocytic proteins in engulfment of *Listeria* and *Rickettsia* protrusions.
— *How are protrusions converted to double membranous vacuoles?* Bacterial protrusions contain two host plasma membranes (figure 5*b*). One of these membranes originates from the human cell receiving the protrusion, and the other is provided by the cell donating the protrusion. Resolution of an internalized protrusion into the vacuole requires scission of both host-derived membranes followed by rejoining (fusion) of these membranes. How scission and rejoining at different membranes are accomplished and coordinated is not understood. A candidate for a human protein mediating scission/rejoining in the cell receiving the protrusion is the GTPase dynamin 2. This GTPase promotes scission during clathrin-mediated endocytosis [178] and is also required for uptake of *Shigella* protrusions [48]. It seems unlikely, however, that dynamin 2 is involved in scission of the membrane from the cell donating the protrusion. Dynamin proteins have never been observed to act within membrane tubules, but only on the outside of these tubules. On the other hand, the mammalian endosomal complex required for sorting (ESCRT) stimulates membrane scission/rejoining from inside tubules to promote multi-vesicular body formation, shedding of membrane blebs and virus budding [179]. It is possible that the ESCRT pathway helps resolve bacterial protrusions by acting in the host cell donating the protrusion. As previously mentioned, human CK-1α is needed for resolution of *Listeria* protrusions [47], and this kinase could potentially regulate ESCRT or other host scission machinery. Alternatively, unidentified bacterial factors might exert membrane scission/rejoining activity in the protrusion-donating cell.

Future work will undoubtedly answer some of the key questions described above. It is likely that high-throughput RNAi-based screens [47] will play an increasingly important role in the identification of host factors involved in cell–cell spread. The choice of mammalian cell line used in such screens, and in the study of bacterial spread in general, is likely to be important. Given the role of cell–cell junctions in controlling dissemination of *Listeria* and *Shigella*, cell lines that have the capacity to develop into polarized monolayers with tight barriers are probably better models for post-ABM steps in spreading than cells that lack these characteristics. Whenever possible, it will also be important to confirm results obtained in tissue culture studies with experiments in animal models. Classic histological studies have proved useful for assessing bacterial spread at a few defined time points [112]. Real-time imaging approaches with live animals [180] are expected to allow more extensive analysis of spreading dynamics *in vivo*. Future work with *Listeria*, *Shigella* and *Rickettsia* will not only help elucidate disease mechanisms, but should also contribute to a better understanding of important aspects of eukaryotic cell biology, including actin assembly, regulation of cell–cell junctions and membrane remodelling.
